# Supplementary material for: A Critical View of the Application of the APEX Software (Aqueous Photochemistry of Environmentally-Occurring Xenobiotics) to Predict Photoreaction Kinetics in Surface Freshwaters
Source: Molecules. 2019 Dec 18;25(1):9. doi: 10.3390/molecules25010009 (PMC7017383; doi:10.3390/molecules25010009)
Supplement: Supplementary file 1 [file molecules-25-00009-s001.zip › 3.2.4_gcc-4.4.0.zip!/3.2.4_gcc-4.4.0/3.2.4_gcc-4.4.0/doc/octave/html/interpreter/Issuing-Warnings.html]

Issuing Warnings - Untitled


Next: Enabling and Disabling Warnings,
Up: Handling Warnings


---

#### 12.2.1 Issuing Warnings

It is possible to issue warnings from any code using the `warning`
function. In its most simple form, the `warning` function takes a
string describing the warning as its input argument. As an example,
the following code controls if the variable a is non-negative,
and if not issues a warning and sets a to zero.

```
     a = -1;
     if (a < 0)
       warning ("'a' must be non-negative.  Setting 'a' to zero.");
       a = 0;
     endif
          -| 'a' must be non-negative.  Setting 'a' to zero.
```

Since warnings aren't fatal to a running program, it is not possible
to catch a warning using the `try` statement or something similar.
It is however possible to access the last warning as a string using the
`lastwarn` function.

It is also possible to assign an identification string to a warning.
If a warning has such an ID the user can enable and disable this warning
as will be described in the next section. To assign an ID to a warning,
simply call `warning` with two string arguments, where the first
is the identification string, and the second is the actual warning.

— Built-in Function: **warning** (template, ...)  
— Built-in Function: **warning** (id, template, ...)  
> Format the optional arguments under the control of the template string
> template using the same rules as the `printf` family of
> functions (see Formatted Output) and print the resulting message
> on the `stderr` stream. The message is prefixed by the character
> string warning: .
> You should use this function when you want to notify the user
> of an unusual condition, but only when it makes sense for your program
> to go on.
>
> The optional message identifier allows users to enable or disable
> warnings tagged by id. The special identifier "all" may
> be used to set the state of all warnings.
> — Built-in Function: **warning** ("on", id)  
> — Built-in Function: **warning** ("off", id)  
> — Built-in Function: **warning** ("error", id)  
> — Built-in Function: **warning** ("query", id)  
> > Set or query the state of a particular warning using the identifier
> > id. If the identifier is omitted, a value of "all" is
> > assumed. If you set the state of a warning to "error", the
> > warning named by id is handled as if it were an error instead.
> >
> >
> >
> > **See also:** warning\_ids.

— Built-in Function: [msg, msgid] = **lastwarn** (msg, msgid)  
> Without any arguments, return the last warning message. With one
> argument, set the last warning message to msg. With two arguments,
> also set the last message identifier.
